# TranslatomeDB: a comprehensive database and cloud-based analysis platform for translatome sequencing data

**DOI:** 10.1093/nar/gkx1034

**Published:** 2017-11-02

**Authors:** Wanting Liu, Lunping Xiang, Tingkai Zheng, Jingjie Jin, Gong Zhang

**Affiliations:** Key Laboratory of Functional Protein Research of Guangdong Higher Education Institutes, Institute of Life and Health Engineering, Jinan University, Guangzhou 510632, China; Chi-Biotech Co. Ltd., Shenzhen 518000, China

## Abstract

Translation is a key regulatory step, linking transcriptome and proteome. Two major methods of translatome investigations are RNC-seq (sequencing of translating mRNA) and Ribo-seq (ribosome profiling). To facilitate the investigation of translation, we built a comprehensive database TranslatomeDB (http://www.translatomedb.net/) which provides collection and integrated analysis of published and user-generated translatome sequencing data. The current version includes 2453 Ribo-seq, 10 RNC-seq and their 1394 corresponding mRNA-seq datasets in 13 species. The database emphasizes the analysis functions in addition to the dataset collections. Differential gene expression (DGE) analysis can be performed between any two datasets of same species and type, both on transcriptome and translatome levels. The translation indices translation ratios, elongation velocity index and translational efficiency can be calculated to quantitatively evaluate translational initiation efficiency and elongation velocity, respectively. All datasets were analyzed using a unified, robust, accurate and experimentally-verifiable pipeline based on the FANSe3 mapping algorithm and edgeR for DGE analyzes. TranslatomeDB also allows users to upload their own datasets and utilize the identical unified pipeline to analyze their data. We believe that our TranslatomeDB is a comprehensive platform and knowledgebase on translatome and proteome research, releasing the biologists from complex searching, analyzing and comparing huge sequencing data without needing local computational power.

## INTRODUCTION

According to the central dogma, all proteins are synthesized via translation process. Therefore, translation is a key regulatory step for all living organisms, linking transcriptome and proteome (1). Previous study has shown computationally and experimentally that the translation control is the most significant regulatory step, whose amplitude is greater than the sum of the mRNA synthesis, mRNA degradation and protein degradation (2). Various mechanisms of translation control (initiation, elongation and termination) influence the protein synthesis and are thus highly relevant to cellular phenotype (3–5).

Two major methods of translatome investigations are RNC-seq (sequencing of ribosome-nascent-chain attached mRNA) (6) and Ribo-seq (sequencing of ribosome protected fragments, RPFs) (7). These two methods reflect different aspects of translatome and cannot be mutually replaced. With these methods, translation initiation efficiency and elongation velocity can be quantitatively assessed using the indices translation ratios (TR) (6) and elongation velocity index (EVI) (8), respectively, both with biological significance. The translatome data also serve as an independent reference for proteome research and was therefore proposed as the fourth key resource pillar of Chromosome-centric Human Proteome Project (C-HPP) (9). It has been intensively used in the C-HPP studies (reviewed in (10–12)) and can facilitate the discovery of new proteins (the proteins encoded by ‘non-coding RNAs’) (13,14).

However, both RNC-seq and Ribo-seq experiments are tricky and cost intensive (especially for Ribo-seq), thus raising request on a comprehensive database to collect up-to-date translatome sequencing data for various species and conditions, and also a unified, convenient, accurate and robust analysis pipeline to integrate previous data and user-generated data.

So far, no previous database includes RNC-seq data, and only a few databases were specifically designed for Ribo-seq. Certainly, these Ribo-seq databases provide valuable online browser, e.g. hosting the RFP and corresponding mRNA data sets in dozens of studies (15). Except data describing and packing, some databases also provide basic analysis and statistic functions, e.g. searching translation initiation codons and the corresponding open reading frames (16); measuring RPF abundance by rpkM (reads per kilobase per million reads) method (17), etc. However, comprehensive and in-depth analyses, especially the inter-dataset comparison analyses, are still lacking for the RNC-seq and Ribo-seq datasets. Thus, it is necessary to establish a systemic database that consummates various translatome sequencing related analysis for mapping, quantifying, differentially expressed gene (DEG) analysis and exploring translation efficiency and elongation velocity with the optimal user experience.

To meet such demand, we established the TranslatomeDB (http://www.translatomedb.net). Thanks to the recent Ribo-seq and RNC-seq developments, we have opportunity to collect 10 RNC-seq, 2453 Ribo-seq and their 1394 corresponding mRNA-seq datasets in the published translatome sequencing datasets for 13 major species including human, mouse, rat, zebrafish and yeast, etc. All datasets can be browsed and searched according to their metadata. Considering the high heterogeneity of the experimental procedures and data quality of these datasets, a unified, accurate and robust analysis pipeline is needed to minimize the bias. Therefore, we used FANSe3 algorithm (the upgraded version of FANSe2 mapping algorithm (18) for cloud computing infrastructure, unpublished yet) to map the raw FASTQ datasets due to its better accuracy, robustness, error-tolerance and experimental verifiability than any other mainstream mapping algorithms (18–21). The TR and EVI values were then calculated for the evaluation of translational initiation efficiency and elongation velocity, respectively. Users can perform DEG analysis between any two datasets of the same species and the same data type. Users can also upload their own FASTQ datasets of their mRNA-seq, RNC-seq and Ribo-seq to perform the same analyses using this database.

## MATERIALS AND METHODS

### Data sources

The TranslatomeDB collects the RNC-seq, Ribo-seq and the corresponding mRNA-seq data from Gene Expression Omnibus (GEO) and Short Read Archive (SRA) databases. The current version includes 2453 Ribo-seq, 1397 mRNA-seq and 10 RNC-seq data sets in 13 species: Arabidopsis, Bacillus, Caenorhabitis, Drosophila, *Escherichia coli*, Human, Mouse, Plasmodium, Rat, Trypanosome, Xenopus, Yeast and Zebrafish (Table 1). Users can upload their own FASTQ datasets of these species to SRA and provide the accession number to the TranslatomeDB for analysis.

**Table 1.** The number of datasets collected in TranslatomeDB

Species	mRNA-seq	Ribo-seq	RNC-seq
Arabidopsis	8	10	
Bacillus	3	7	
Caenorhabditis	30	31	
Drosophila	14	24	
*E. coli*	30	89	
Human	186	353	7
Mouse	779	1125	
Plasmodium	10	10	
Rat	8	8	
Trypanosome	18	18	
Xenopus	3	3	
Yeast	147	285	3
Zebrafish	161	493	

### Data analysis pipeline

SRA files were fetched from NCBI SRA database and converted to FASTQ format by SRAToolkit v2.8.2 with the parameter *–split-files*. The barcode or index part of each read were trimmed.

mRNA-seq, RNC-seq and Ribo-seq reads were mapped using FANSe3 against transcriptome reference sequences (Supplementary Table S1). When mapping RPFs, the first 23 bases of each read were used to avoid the possible remains of linker or adaptor sequences. This length was chosen to unify the analysis of prokaryotic and eukaryotic RPFs, whose average lengths are 24 and 28 nt, respectively (7,8,22). Only one error (mismatch or indel) was allowed in the mapping, in consistence with previous approaches (17). The seed length was set to 10. Under such settings, the best alignment within the error tolerance can be found with mathematical guarantee (18,19). When mapping RNC-seq and mRNA-seq datasets, we allowed 6% error of the read (23–25) due to the variable read length of different datasets. In case of paired-end reads, only the first end was taken for mapping because this will not influence the quantification (26). Splice variants were merged. Quantified genes (with >10 mapped reads) (27) were quantified using rpkM method (28).

Pairwise DEG analysis can be done between any two datasets in the same species and dataset type, e.g. SRR768268 and SRR611122 are both RNC-seq datasets of human. The DEG analysis were performed by edgeR package (29), because edgeR is experimentally proven to be superior to the other DEG analysis tools on minimized false positives and false negatives (30). The result was visualized using an interactive volcano plot and a downloadable table including log fold change and *P*-value. Translational efficiency (TE), TR and EVI were calculated using the multiple types of sequencing on one sample (6–9):

\begin{equation*}{\rm{TE}} = \frac{P}{M}\end{equation*}

\begin{equation*}{\rm{TR}} = \frac{R}{M}\end{equation*}

\begin{equation*}{\rm{EVI}} = \frac{{{R^2}}}{{M \cdot P}}\end{equation*}

where R, M, P represents the rpkM values of RNC-seq, mRNA-seq and Ribo-seq from a single sample, respectively.

**Figure 1.**
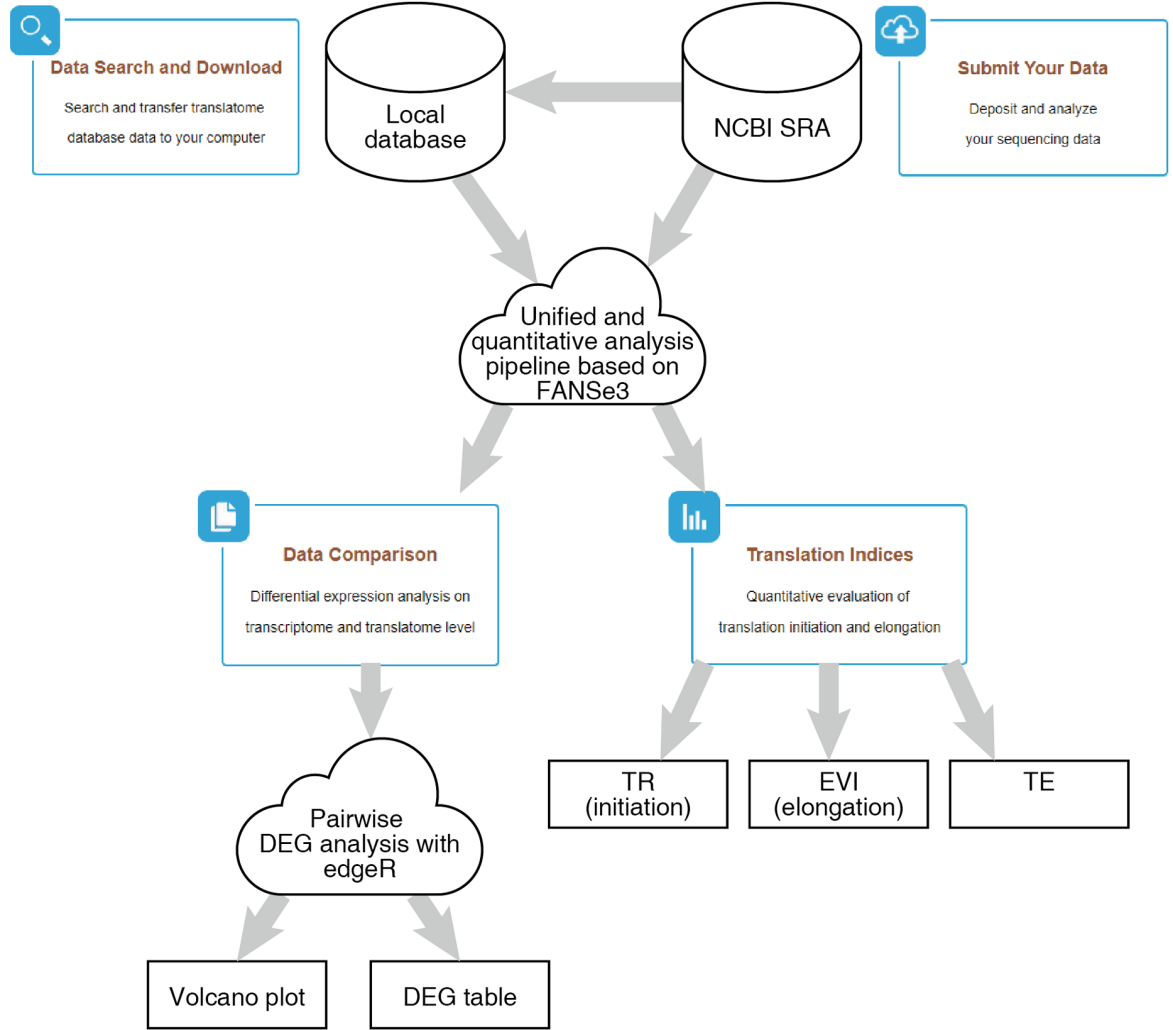
Functional flow chart of TranslatomeDB. The cloud symbols denote the computational-intensive steps that are performed in the Chi-Cloud NGS analysis system, which do not need any local computational resource.

The data analysis pipeline is illustrated in Figure 1.

## RESULTS

### There are four functional sections of TranslaomeDB

#### Data search and download

Users can search the sequencing datasets using nine fields of the metadata, including species, dataset types, GEO numbers, sequencing instrument, cell line/tissue types, SRA accession number and so on. One or more key words are accepted to precisely get the desired datasets. Clicking the ‘Details and download’ icon will direct the users to the detailed meta information (automatically fetched from SRA database) of the dataset including the hyperlink to the corresponding articles and a direct FTP download link of the raw sequencing dataset. Alternatively, users may also browse datasets by clicking on the species list on the left side of the homepage, or type in key words into the ‘quick search’ box on the top of the homepage. Search results can be further refined by typing in more keywords in the other fields. In the ‘Details info’ page of each dataset, the gene quantification list, including read count, rpkM and detailed gene information, is provided, which can be browsed and searched.

#### Data comparison

**Figure 2.**
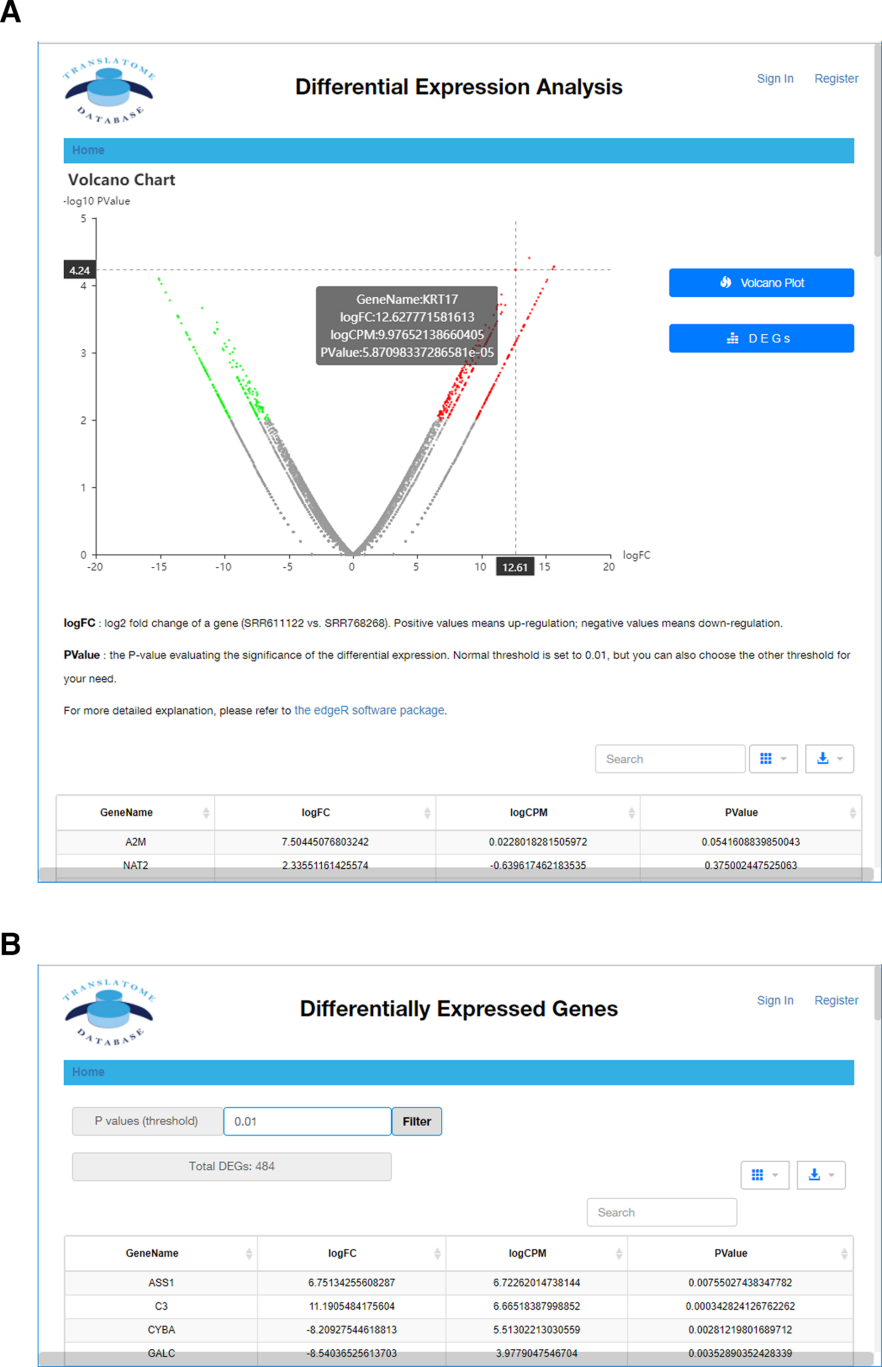
Differentially expressed gene (DEG) analysis. (**A**) The interactive volcano plot of a typical DEG analysis (RNC-seq datasets of human H1299 and HBE cell lines). Detailed gene information is shown when mouse moves over a dot. Full DEG result list are shown at the bottom of the page. (**B**) Given a *P*-value threshold (by default 0.01), the TranslatomeDB filters the significant differentially expressed genes (DEGs) under this threshold. The list can be easily searched and downloaded.

This section performs DGE analysis of any two datasets of the same species and same data type (e.g. two RNC-seq datasets, two Ribo-seq datasets, or two mRNA-seq datasets). Therefore, the regulation in transcriptional level and translational level can be quantitatively evaluated, respectively. Select two datasets and click ‘Compare’ button to compare the metadata for the user to confirm that these two datasets are of his/her interest. For the user's convenience, the same attributes of both datasets were merged and marked as grey and may be also hidden. Then, clicking the ‘Differential Expression Analysis’ will perform the DGE analysis using edgeR software package due to its superior performance on minimized false positives and false negatives (30). The calculation is performed in the cloud computing infrastructure within seconds and do not need any local computational resource of the user. An interactive volcano plot is automatically generated. Mouse hover on a dot of interest will trigger the display of detailed gene name, log_2_ fold change (logFC) and *P*-values represented by this dot (Figure 2A). A detailed list of the gene name, logFC and *P*-values of all genes (not only the significantly DEGs) are listed as text tables. All columns can be sorted and all fields can be searched, for example, typing in the gene name in the ‘Search’ box will quickly locate the desired gene and its corresponding values. The table can be downloaded as six common formats, including plain text table, csv, xml and Microsoft Excel format, for further analysis or plotting. Clicking ‘DEGs’ button triggers an interactive table that can filter the DEGs based on user-defined *P*-value. This facilitates users to export the DEG list for further gene ontology and pathway analyses (Figure 2B).

#### Translation indices

**Figure 3.**
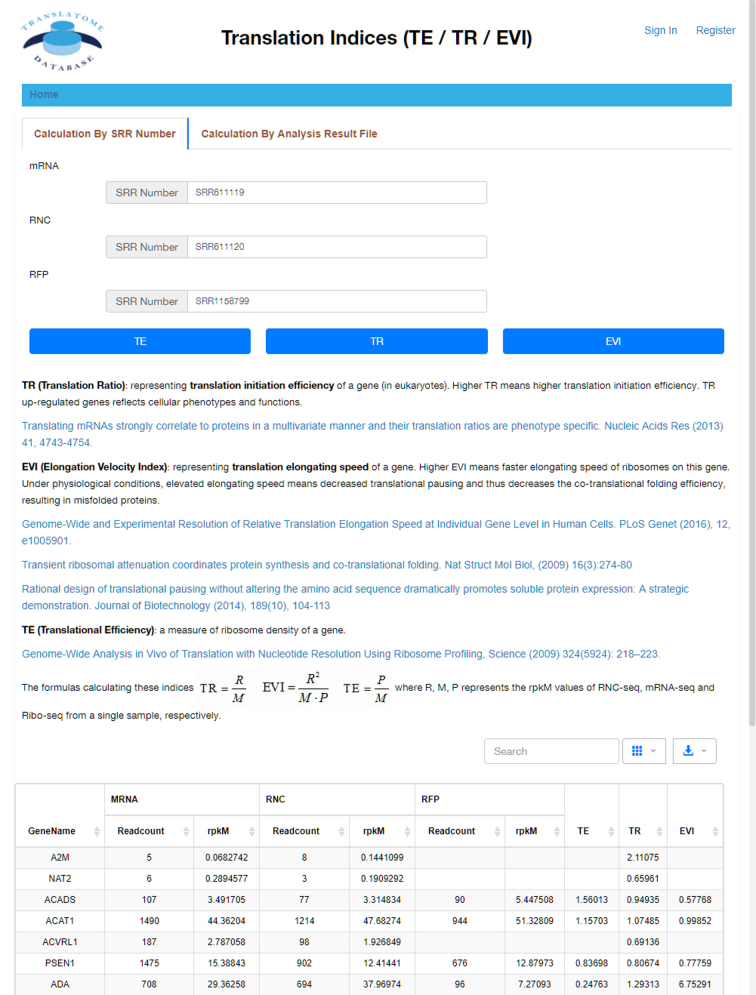
Translation indices calculation. Selecting 2–3 datasets (mRNA-seq, RNC-seq and Ribo-seq) for one sample allows the TR, EVI and TE calculation to quantitatively assess the translation initiation efficiency and elongation speed, respectively. Results are displayed in table. This table is searchable and downloadable.

This section performs the calculation of the translation indices TE, TR and EVI, which quantitatively represents the translational initiation efficiency and elongation velocity. TE is calculated by the Ribo-seq data and the corresponding mRNA data to contribute the dynamic range of gene expression; TR can be calculated using the mRNA-seq and the corresponding RNC-seq data to reflect cellular functions and phenotypes; EVI can be calculated using the mRNA-seq, the corresponding RNC-seq and Ribo-seq data to reflect protein co-translational folding efficiency. Since TE, TR and EVI are calculated based on rpkM values of each gene in different sequencing types, the corresponding rpkM values are also listed (Figure 3). Similar to the *Data Comparison* section, the list can be searched and downloaded in six formats.

#### Submit your data

Users can submit their own translatome sequencing datasets (including RNC-seq, Ribo-seq and corresponding mRNA-seq) for analysis. To do so, users need to submit their FASTQ datasets to NCBI SRA (https://www.ncbi.nlm.nih.gov/sra) and get the publicly available SRA FTP URL, then click *Submit* page of TranslatomeDB, register or log in into the user account, fill in the required information including SRA FTP URL. After submitting all these information, the TranslatomeDB will automatically fetch the data from SRA and start processing the data using the unified pipeline (see ‘Materials and Methods’ section). The computational intensive mapping and quantification process is solely done in the Chi-Cloud, the supercomputer-based cloud NGS analysis system developed and operated by Chi-Biotech Co. Ltd. Therefore, the users simply need just one-click without any powerful workstations or bioinformatics knowledge for all these analysis process. Users may log into their account to check the progress of data processing, as the data fetch and mapping takes some time. The results will be stored in the TranslatomeDB under the user’s account and thus can be searched and analyzed in all other sections. In the ‘My Data’ page, users can manage their datasets.

To keep the consistency and comparability of datasets across the studies, the reference sequences should be uniform for one species. Therefore, the TranslatomeDB currently only allows the upload and analysis of the 13 species listed in the homepage. We will include more species in case of more model organisms were investigated on the translatome level. The users may also request specific species via the feedback function.

## DISCUSSION

Growing interest in translatomics requests more translatome sequencing data. However, the difficult and challenging experimental procedures of translatome sequencing and its corresponding high cost largely restricted the application of these methods, especially under the current status that the commercialized experimental service is hardly available. Therefore, mining valuable information from the published data would help biologists to briefly validate their intuitive ideas using the previous data before investing much time, effort and cost on such direction. Unified, accurate and robust analysis pipeline reinforces the comparability between different studies, as well as user-submitted sequencing datasets. All the computation does not need local computational power and can be accomplished within a few clicks. This is especially convenient for the biologists lacking bioinformatics knowledge, skills and hardware.

Compared to the current RPF-related databases, there are several unique advantages of our TranslatomeDB: (i) more comprehensive collection including RNC-seq, Ribo-seq and mRNA-seq datasets; (ii) much more datasets collected; (iii) offers DGE analysis to compare two datasets, even from two studies; (iv) calculates TR and EVI to quantitatively assess the translation initiation efficiency and elongation speed; (v) one-click, cloud-based analysis of user-uploaded datasets without local computational resources or bioinformatics skills. TranslaomeDB is designed mainly to quantitatively evaluate the global translation. Therefore, it does not pile up RPF reads to predict individual translation initiation and termination sites. For these purposes, RPFdb (17) and GWIPS-viz (15) would be better choices. The comparison of the above-mentioned three databases are listed in Table 2.

**Table 2.** Feature comparison of TranslatomeDB, RPFdb and GWIPS-wiz

	TranslatomeDB	RPFdb (17)	GWIPS-wiz (15)
Data type	mRNA-seq, RNC-seq, Ribo-seq	Ribo-seq only	mRNA-seq, Ribo-seq
Datasets collected (till 9 August 2017)	3857	777	N/A
Species	13	8	N/A
Aligner	FANSe3 (most accurate)	STAR	bowtie (least accurate)
Meta information	searchable and comparable	searchable, not comparable	not searchable
Gene-wise rpkM	yes	yes	no
DGE	yes	no	no
Translation initiation and elongation indices	yes	no	no
Analyze user-uploaded datasets	cloud-based, one-click	no	no
RPF reads pile-ups		yes	yes

To keep the database up-to-date, we will be updating the content in two ways: (i) we will repeat literature search and supplement the newest translatome-related datasets one to two times per year or more frequently; (ii) we will integrate the user-uploaded and analyzed datasets into our main database.

Since a major function of TranslatomeDB is the processing of large and raw sequencing datasets, which is very computationally intensive, the computational cost partly determines the long-term sustainability. Previous tests showed that FANSe2 (for links, see Supplementary Table S2) is a very efficient mapping algorithm compared to the other mainstream algorithms (18,21). To fully utilize the high-performance cloud computing infrastructure with many (48+) cores and huge memory, we developed the upgraded version FANSe3 algorithm (for links, see Supplementary Table S2) that is almost 30-folds faster than FANSe2 in RNA-seq applications, and the RNA quantification results are almost identical (Pearson *R* > 0.999). An example of the dataset SRR1257177 can be found in Supplementary Figure S1. Therefore, the FANSe3 can replace FANSe2 and enables to process hundreds of datasets within 1 day per server node. Considering the large number of server nodes in the commercialized Chi-Cloud, processing such data barely burdens the company. In fact, mapping all 3857 datasets that are currently collected in the TranslatomeDB only occupied 228 CPU hours, which is less than 10% of the daily capacity of the current Chi-Cloud system. The computation of DGE and translational indices is accomplished in seconds, and the bottleneck would be the network transfer. The light load indicates that this commitment can be easily increased with the expansion of the translatome research and the growth of the company. As a backup, a smaller Chi-Cloud backup system configured by the Chi-Biotech Co. Ltd. will be placed in the Jinan University, Guangzhou, China, which is funded by the university and national grants (see the funding section). All these factors facilitate the long-term maintenance of the TranslatomeDB.

We believe that our TranslatomeDB is a comprehensive platform and knowledgebase on translatome and proteome research, releasing the biologists from complex searching, analyzing and comparing huge sequencing data without needing local computational power.

## Supplementary Material

Supplementary Data
