# Supplementary material for: TranslatomeDB: a comprehensive database and cloud-based analysis platform for translatome sequencing data
Source: Nucleic Acids Res. 2017 Nov 2;46(Database issue):D206–12. doi: 10.1093/nar/gkx1034 (PMC5753366; doi:10.1093/nar/gkx1034)
Supplement: Supplementary Data [file gkx1034_supp.pdf]

TranslatomeDB: a comprehensive database and cloud-based analysis platform for translatome sequencing data

Wanting Liu [*,1], Lunping Xiang[*,2], Tingkai Zheng[*,1], Jingjie Jin, Gong Zhang[#,1,2]

Supplementary Materials

Table S1: The details of transcriptome reference sequences

Species	Source *	Version
Arabidopsis	NCBI	TAIR10 RefSeq-RNA
Bacillus	NCBI	str.168 RefSeq-RNA
Caenorhabditis	UCSC	WS220/ce10 RefSeq-RNA
Drosophila	UCSC	Dm6 RefSeq-RNA
E.coli	NCBI	BW25113 RefSeq-RNA
Human	UCSC	hg19 RefSeq-RNA
Mouse	UCSC	mm10 RefSeq-RNA
Plasmodium	NCBI	3D7 RefSeq-RNA
Rat	UCSC	Rn6 RefSeq-RNA
Trypanosome	NCBI	DAL972 RefSeq-RNA
Xenopus	NCBI	Xenopus_laevis_v2 RefSeq-RNA
Yeast	NCBI	S288C RefSeq-RNA
Zebrafish	UCSC	Zv9/danRer7 RefSeq-RNA

* Download source: UCSC = UCSC Genome Browser; NCBI = NCBI Nucleotide database.

Table S2: The websites of FANSe2 and FANSe3 algorithms:

**FANSe2**	http://bioinformatics.jnu.edu.cn/software/fanse2/<br>http://www.chi-biotech.com/fanse2
**FANSe3**	http://www.chi-biotech.com/fanse3

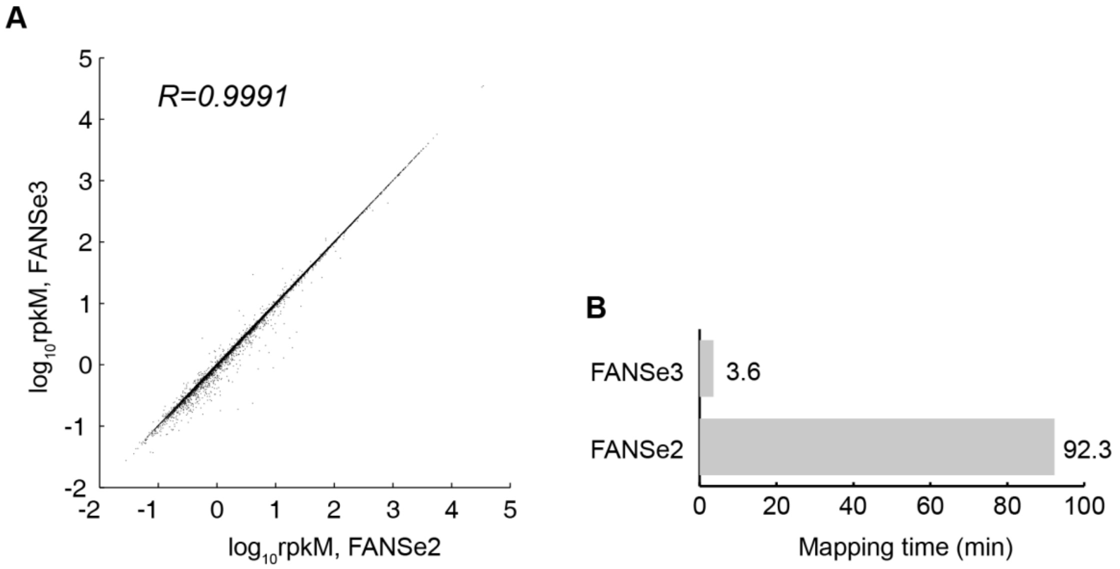

Figure S1: Comparison of FANSe3 and FANSe2 when mapping the dataset SRR1257177. (A) RNA-seq quantification result. (B) Running time of the mapping procedure. The test was performed in a dual E5-2680V2 server node (20 cores, 40 threads) with 192GB RAM installed.
